# Microbiomes in Suspended Soils of Vascular Epiphytes Differ from Terrestrial Soil Microbiomes and from Each Other

**DOI:** 10.3390/microorganisms9051033

**Published:** 2021-05-11

**Authors:** Alen K. Eskov, Alexei O. Zverev, Evgeny V. Abakumov

**Affiliations:** 1 Faculty of Biology, Lomonosov Moscow State University, 1–12 Leninskie Gory, 119991 Moscow, Russia; 2 Tzitzin Main Botanical Garden, Russian Academy of Sciences, 4 Botanicheskaya ul., 117628 Moscow, Russia; 3 Faculty of Biology, Saint-Petersburg State University, 7/9 Universitetskaya nab., 199034 St. Petersburg, Russia; azver.bio@gmail.com (A.O.Z.); e_abakumov@mail.ru (E.V.A.); 4 All-Russian Research Institute for Agricultural Microbiology, 3 Podbelsky chausse, 196608 St. Petersburg, Russia

**Keywords:** epiphytes, trophic relations, suspended soils, microbiome, metagenomic analysis, Vietnam

## Abstract

Microbial biodiversity parameters for tropical rainforests remain poorly understood. Whilst the soil microbiome accounts up to 95% of the total diversity of microorganisms in terrestrial ecosystems, the microbiome of suspended soils formed by vascular epiphytes remains completely unexplored. Samples of ground and suspended soils were collected in Cat Tien National Park, southern Vietnam. DNA extraction and sequencing were performed, and libraries of 16s rDNA gene sequences were analyzed. Alpha diversity indices of the microorganisms were the highest in the forest ground soil. In general, the microbiological diversity of all the soil types was found to be similar at the phylum level. Taxonomic composition of the bacterial communities in the suspended soils of plants from the same species are not closer than the taxonomic compositions of the communities in the suspended soils of different plant species. However, the beta diversity analysis revealed significant differences in the movement of mineral elements in terrestrial versus suspended soils. Our data showed that the suspended soils associated with vascular epiphytes were a depository of unique microbiological biodiversity. A contributing factor was the presence of large amounts of organic matter in the suspended soils—deposits collected by the epiphytes—which would have been degraded by termites if it had reached the ground. Further, the nutrient content of the suspended soils was prime for soil respiration activity and taxonomic microbial community biodiversity.

## 1. Introduction

One of the most important structural components of tropical forests are epiphytes. Vascular epiphytes are, in the edaphic sense, plants with no access to traditional native soil and its nutrients [1]. The extent of this isolation is still poorly understood [2]. The existing data on the mineral nutrition and organic-matter deposition of different epiphyte ecomorphological groups [3,4,5] and habitats [6] are quite contradictory.

Many epiphytes are characterized by their roots’ ability to accumulate significant volumes of organic substrates, known as suspended soils [1,7]. Suspended soils can be defined as organic matter that has not fully decomposed and has not turned into the humus typical of terrestrial soils [8]. This definition assumes that the source of the organic matter is exclusively phorophyte litter. However, several facts contradict this understanding of suspended soils. Phorophyte leaf litter is intercepted by branches but is carried away in a very short period of time [9]. The remaining organic matter amounts to a very small fraction of epiphyte biomass production. In contrast, much of the suspended soil’s organic matter is preserved throughout the life of the epiphyte, decomposing only if the epiphyte falls to the ground [10]. These findings indicate that the crown nutrition cycle and epiphytic nutrition are largely separate from wood litter [4]. By contrast, our previous studies have suggested that complete humification processes do occur in suspended soils [11,12]. Therefore, the edaphic role of epiphytic substrates remains underestimated.

The important ecosystem role of suspended soils can be explained by the scale of the epiphyte eco-group itself. Approximately 10% of all vascular plants are epiphytes [7,13]. Epiphytes deposit significant fractions of organic matter into the crowns of rainforest trees. The green epiphytic biomass can be compared to the green leaf biomass of broad-leaved trees [14,15,16], although the most weighted estimates show that the green epiphytic biomass does not exceed 10% of the green-leaf biomass of broad-leaved trees [7]. The mass of epiphytic material as a whole (green biomass + suspended soils) is much higher than the green biomass of epiphytes alone. Epiphytic substrates provide shelter to many small animals, which significantly contributes to the structural and spatial biodiversity of the rainforest. Epiphytic materials are home to many invertebrates; for example, microarthropods [17,18], including Collembola and Oribatidei [19]. The biomass of suspended-soil inhabitants is 20 times greater than that of the most inhabited soil horizon [20]. In several *Asplenium nidus* ‘trash-can’ epiphytic fern specimens collected from three Vietnamese sample locations, 150 species of invertebrates were found [20]. The biodiversity of ecosystems found in tropical soils is not as great as that found aboveground ecosystems; additionally, the diversity of the soil fauna [21] and prokaryotic communities [22] found in tropical soils is less than that found in temperate soils. Tropical soils, during deforestation to natural forests, are often prone to degradation and loss of fertility. Following deforestation, a radical reorganization of the taxonomic structure and diversity of soil microbial communities takes place [23,24,25].

The biodiversity of prokaryotes in suspended soils remains a completely unexplored area. At first glance, trends in prokaryotic microbiomes appear to repeat the known patterns of invertebrates [17,18,19]. However, in this case, only terrestrial soils are a natural source of microbiome biodiversity. Therefore, we posited that, due to the unique individual genesis observed in each suspended soil community (often associated with the epiphyte itself), each suspended soil’s microbiome would be very different from those of terrestrial soils, disturbed soils, and other suspended soils. This trend should be clear when observing the beta diversity, elucidated by the actualization of edaphic differences. To test this hypothesis, we studied suspended soils formed by several species of primary- and artificial-forest (plantation) epiphytes, as well as undisturbed and disturbed soils in Cat Tien National Park, South Vietnam. This place is one of the most unique regions for biodiversity protection. During the American–Vietnamese war (1961–1971), the mountainous forest of Cat Tien and adjacent regions, including Ma-Da Forest (Cat Tien and Ma-Da form one giant forested area) was intensely affected by defoliants and herbicides, particularly Agent Orange, and by napalm fires [26,27]. Later, the remaining forests of Dong-Nay were clear-cut up until 1998 [27]. Cat Tien has avoided clear-cutting because of its designation as having national park status. By the end of the 20th century, this national park was represented by a mixture of various forest stands, with evidence of intensive anthropogenic disturbance [26]. In 1996–1998, intensive reforestation measures were undertaken, in the context of forest restoration processes, on 200 ha of territory [28,29]. In 2001, Cat Tien was included in UNESCO’s list of biosphere reserves [27]. Thus, our study comprised an investigation of the microbiome of this benchmark forest, which faced serious anthropogenic impacts in the previous century, but has been reforested in the last 20 years.

## 2. Materials and Methods

### 2.1. Field Studies

Field studies were conducted in southern Vietnam in 2018. The habitat investigated was a lowland forest. Cat Tien National Park is located in the lowlands of Dong Nai Province. The territory of the national park is affected by monsoons and is covered primarily by deciduous forests and, to a lesser degree, disturbed forests [26]. The subequatorial monsoon climate of the national park is characterized by two distinct seasons—wet/rainy (May–October) and dry (November–April). The annual precipitation is 1800–2500 mm and the average annual temperature is 26.2 °C [30]. Cat Tien’s forests grow on rich soils layered with volcano-sedimentary debris, which are classed as Folic and Hyperskeletic Vertisols [31].

### 2.2. Sample Collection

Three samples were collected from each of two sample sites. The soils were classified according to the World Reference Base international standard [32]. The sites were:

(1). The bank of the Mixed primary forest in the bank of the Dong Nai River (11°25′55” N, 107°25′48” E). A set of ground soil samples was taken from the upper horizon of a Skeletic Greyzemic Umbrisol (Clayic) soil (FG), and two sets of epiphytic suspended-soil samples were taken from *Drynaria quercifolia* (FS2) and *Vittaria* sp. (FS1) specimens, respectively; and
(2). Artificial habitats in a mixed tropical tree plantation (11°24′05” N, 107°22′29” E). Loamy terrestrial soil samples were taken from the top of the AU horizon of 25-year-old grey-humus lithozems (PG). Two suspended soils were taken from *D. quercifolia* (PS1) and *Lemmaphyllum microphyllum* (PS2) respectively.

The species of epiphytes from which we collected the suspended soils were typical of tropical Southeast Asia. *Drynaria quercifolia* (Polypodiaceae) is a typical ‘trash-can’ epiphyte, accumulating significant masses of organic matter in their ‘nests’. *Vittaria* sp. (Pteridaceae) forms more flattened masses of suspended soils under its roots, adjacent to the phorophyte cortex. *Lemmaphyllum microphyllum* (Polypodiaceae) has the most specific ecomorphology—settling on relatively thin branches, it forms extensive ‘beards’ out of the plexus of rhizomes, trapped leaves, and decayed remains of branches and nests of dendrobiont ants that accumulate there. One specimen can completely cover a tree, preventing dead branches and leaves from falling to the ground. *Lemmaphyllum microphyllum*’s method of depositing dead organic matter is similar to the formation of ‘suspended litter’ by mushroom rhizomorphs in a tall forest. Therefore, the suspended soils associated with this species are less decomposed.

Since the forest is partly seasonally flooded, we chose the sampling sites near the river so that the ground soils in both the primary forest and the plantation would spend part of the year in a flooded state. For collection of the suspended soils, trees with a well-developed and not highly located epiphytic community were selected. Each sample was collected from a different tree. The epiphytes chosen were adults and well developed specimens. They were picked using a stick with a hook on the end at a height of 5–6 m, on average. The suspended soils were separated from the plant debris and roots, sifted through a sieve, and mixed. Thus, each sample was a mixed sample of the entire clod of suspended soil of one epiphyte specimen. The ground soils were collected under the trees from which the epiphytes were collected. Litter and small plant debris was removed from the ground, a shallow hole was dug, and the sample was taken from a depth of 10–15 cm. Soil samples (50 g) were collected from five points at each site using the ‘envelope method’ (four samples from the corners of a square with sides of 1 m and one from the center). The suspended soils samples also weighed 50 g. Thus, each suspended soil sample was the soil of one epiphyte, and the ground soil was a mixture of five points per 1 square meter at the base of the tree on which the epiphytes grew—six samples in total, three from two habitats. Each sample was divided into two parts. Most (49 g) was dried in paper bags and subsequently subjected to routine soil analysis. The second part was subsampled to provide an aliquot of 0.2 g, which was homogenized and stored at −70 °C in dry ice; this was used for DNA extraction in the laboratory.

### 2.3. Routine Soil Analysis

The dried samples were passed through a 2 mm sieve. Samples were selected from each horizon and analyzed for their physical and chemical properties. The analyses were conducted in the certified laboratory of St. Petersburg State University in the Department of Applied Ecology, Russia. The soils were analyzed according to the following methods: (1) the actual acidity (pH_H2O_) was determined using a stationary pH meter in an aqueous solution; (2) the soil microbiological activity (substrate-induced basal respiration) was determined using incubation chambers [33]; (3) the carbon and nitrogen contents were determined via the ‘dry combustion’ method using a C-H-N-analyzer; (4) the texture class was identified via the Kachinsky sedimentation method, which is based on the separation of various size fractions in a pyrophosphate peptonized water column, where aliquots of each fraction were taken from different depths, the residue was dried by evaporation in a ceramic cup, and further gravimetric evaluation was made on the contents of each fraction [34]; (5) the carbon content of the microbial biomass (C_mic_) was determined using moist field samples via the chloroform fumigation–extraction method; (6) the metabolic quotient (the ratio of the respiration of C–CO_2_ to C_mic_ per day of incubation) was calculated [35]; and (7) the soil microbiological characteristics of all the soil horizons were determined, when there was enough soil left. Because the soil respiration and microbial biomass were measured under laboratory conditions, the data obtained could not be interpolated directly to the field conditions, but it could be used for a comparison of the soil microbiological activity under the same experimental conditions (temperature 20 °C, moisture 60%) [36].

While the pH in a water suspension is normally used for the determination of easily soluble protons in soil solutions, the potential acidity is used for the characterization of the exchangeability of the soil. Thus, the exchange acidity was also investigated. The exchange acidity is determined by the hydrogen and aluminum ions present in an exchange state in the soil’s absorptive colloidal complex. While the colloidal complex has a different capacity, the amount of cations, especially protons, which represent the potential acidity is of special interest in the assessment of total soil acidity. Exchangeable ions can be extracted from soil via a neutral salt solution. Normally, a calcium chloride solution is used to determine the exchange acidity of soils (the ratio being 1:25 for organic horizons and 1:2.5 for mineral soils). The exchange acidity was measured by taking the pH of the salt extract (pH_CaCl2_). Hydrolytic acidity is caused by the hydrogen and aluminum ions present in the exchangeable (or partially non-exchangeable) state of the soil absorptive colloidal complex. These ions are extracted via a solution of the hydrolytically alkaline salt of a strong base and a weak acid (usually a 1-N solution of sodium acetate). The hydrolytic acidity is the sum of the exchange and actual acidity [37]. Thus, if there are any differences between the exchangeable and actual acidity in any soil, it can be concluded that there is a colloidal complex present that can absorb not only the protons, but other positively charged cations, which is important for potential evaluation of the nutritional state of a soil substrate.

### 2.4. Metagenomic Analysis

The total soil DNA was isolated from the previously prepared samples using an MN NucleoSpin kit (MN, Germany). The homogenization of the samples required by one of steps in the protocol was performed using a Precellys 24 homogenizer (Bertin Technologies, Montigny-le-Bretonneux, France), at 6500 rpm for 30 s, twice. The extraction quality was estimated by electrophoresis in agarose gels (1% *w*/*v* in tris-acetate-EDTA), followed by a polymerase chain reaction ability trial (presence of PCR product after PCR with required primers).

Amplification of the V4 variable region of the 16S rRNA gene was carried out using the F515 (GTGCCAGCMGCCGCGGTAA) and R806 (GGACTACHVGGGTWTCTAAT) primers [38] and the Illumina protocol, *16S* Metagenomic Sequencing Library Preparation [39]. The libraries were sequenced using an Illumina MiSeq with paired-read lengths of 2 × 250 bp. Sequencing was performed using equipment of the Core Centrum ‘GenomicTechnologies, Proteomics and Cell Biology’ in ARRIAM”.

Processing of the sequence data was carried out using R v.3.6.3 software, and the dada2 (v.1.14.1) [40], phyloseq (v.1.30.0) [41] and DESeq2 (v.1.26.0) packages [42]. Taxonomic annotation was performed using the SILVA database, v.132 [43]. The chloroplast and mitochondrial gene sequences were deleted from the dataset. The amount of reads per sample were between 5531 and 20,268, the mean value being 15,776 reads per sample. For the best performance in the downstream analysis, a threshold value of 6000 reads per sample was set.

Before the diversity estimation, the operational taxonomic unit (OTU) table was normalized to 6118 reads per sample (the minimal abundance from all samples after the threshold filter) via rarefaction. Diversity within the communities (alpha diversity) was estimated by calculating the observed OTU number, and the Simpson and Shannon indices. Differences between communities (beta diversity) were estimated using the metrics of the Bray–Curtis dissimilarity. For estimating the scale of influence of the sampling site on the microbial communities, PERMANOVA factor analysis (vegan v.2.5–6 R package) was used. The Mantel test (also from vegan package) was used to estimate the correlation of the beta diversity and the nutrition component composition.

We performed differential abundance analyses between the samples. DeSEQ2 was employed to make pairwise comparison of the samples. Based on the results, all the OTUs could be classed in one of two groups—‘variable’ if the OTU abundances were significantly variable between groups of samples, according to the DeSEQ analysis, or ‘common’ if they were not. Moreover, we provided taxonomical information about the OTUs, where the relative abundance increased (or decreased) more than 10 times.

## 3. Results

### 3.1. Soil Nutrition Properties

All the soils investigated had acidic pH values (Table 1). The levels of basal soil respiration were not high, which is evidence of a stabilization of the organic-matter mineralization processes. The total organic carbon content was higher in the suspended and native ground soils than in the disturbed ground soil. The only difference between the ground and suspended soils was that the C/N ratio was higher in the suspended soils (*t*-test, *p* < 0.04). This indicates lower mineralization rates in the suspended soil in comparison with the ground soils. The nutrient content was quite variable in the soils (Table 1). The calculated Spearman’s correlation coefficient (Supplementary Table S1) showed that the basal respiration values strongly reflected available phosphorous, potassium and ammonium, which is typical of all organo-mineral soils. There was no essential correlation between the levels of basal respiration and bulk (total) organic carbon and nitrogen. This was because the bulk carbon and nitrogen content presented in an easily mineralizable form. In general, the described level of microbiological activity corresponded well with the copiotrophic strategy of the microorganisms’ metabolisms.

### 3.2. Terrestrial and Suspended Soil Microbial Diversity

We focused on the differences between the microbial communities from the different types of suspended soils (PS1, PS2, FS1, and FS2) and native soils from the forest (FG) and plantations (PG). All the samples had three for FS2 and four replications of individual subsamples for all others samples.

**Figure 1:**
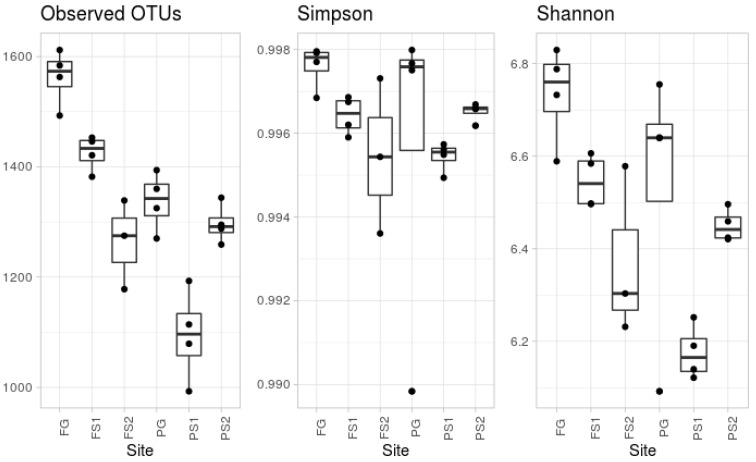
Measured alpha diversity metrics.

According to the alpha diversity metrics (see Figure 1), there was a significant difference in the observed OTUs. The most diverse, according to the number of observed OTUs, was the forest ground soil (FG). The microbial community from the other ground soil, from the plantation (PG), was significantly less diverse, in terms of observed OTUs. This group contained an outlier sample, according to Simpson’s evenness index. According to the observed OTU index, the suspended soils were less diverse than the ground soil from the same location, whereas Simpson’s index did not show any significant difference.

**Figure 2:**
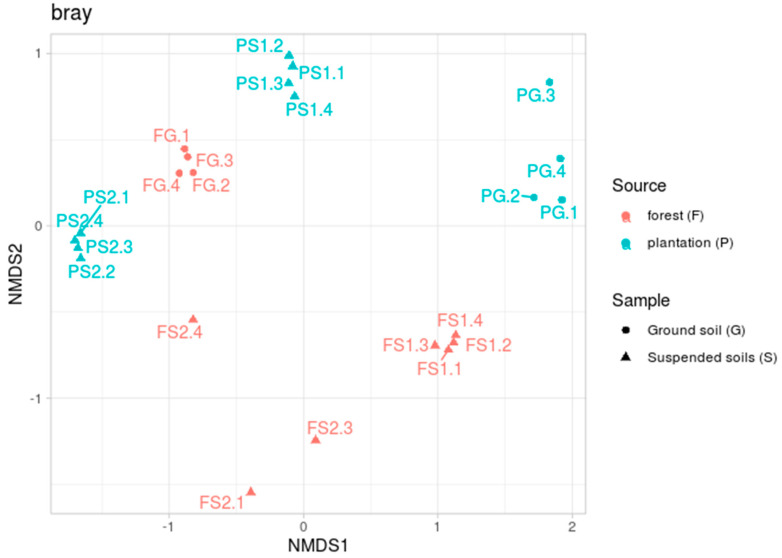
Beta diversity based on the Bray–Curtis dissimilarity metric, NMDS ordination.

The beta diversity analyses, performed by calculating Bray–Curtis dissimilarity indices, showed a complicated effect (see Figure 2). The microbiomes of the forest ground samples (FG) located in a tight cluster, whereas the ground soil samples from the plantation (PG) were more dispersed. Tight clusters were also formed by both the suspended soils from the plantation (PS1 and PS2) and one of the forest suspended soils (FS1). The other suspended soil sample (FS2) from the forest, by contrast, was spread by a plot.

The PERMANOVA results demonstrated the significance of both factors—the source of the samples (forest or plantation) and their location (terrestrial or suspended)—with *F*_1_ = 3.64, *n*_1_ = 1, *p*-value_1_ = 0.01, *F*_2_ = 3.61, *n*_2_ = 1, *p*-value_2_ = 0.02 and *n*_3_ = 20.

**Figure 3:**
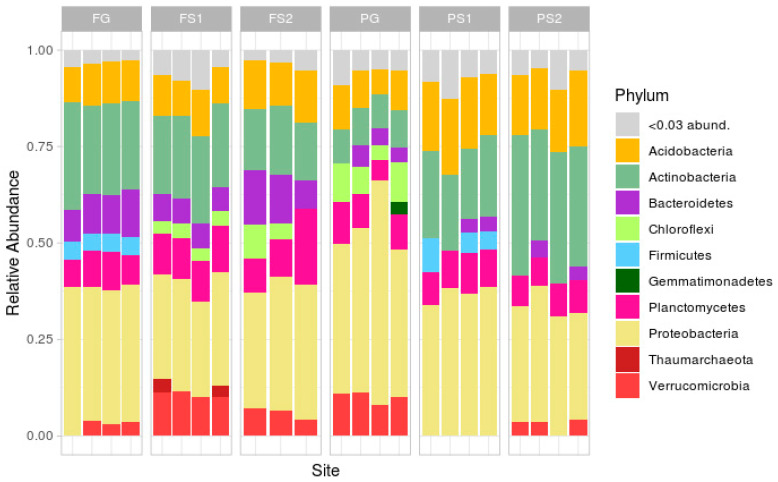
Taxonomic structure of samples at the phylum level.

Taxonomic analysis at the phylum level (Figure 3; rare phyla (less than 3%) were masked in the ‘<0.03 abund.’ group) demonstrated a classical soil composition, with the main part of the reads being attributed to Proteobacteria, followed by Actinobacteria, Acidobacteria, Bacteroidetes, Planctomycetes and Verrucomicrobia.

In the forest site, the soil microbiome compositions were similar, the differences being in the relative abundances of Verrucomicrobia (more in FS1, less or absent in FG), Firmicutes (absent in FS1 and FS2) and Chloroflexi (absent in FG). In the plantation site, the compositions were less similar, with differences in the abundance of Verrucomicrobia (absent in suspended soils PS1 and PS2), Chloroflexi (only present in the PG samples), Actinobacteria and Acidobacteria (both more frequent in PS1 and PS2), and Firmicutes (present in PS1, not in PS2 or PG).

Comparison of the two terrestrial soils showed several differences, mainly in the phyla Actinobacteria, Bacteroidetes, Firmicutes, Chloroflexi, Proteobacteria, and Verrucomicrobia. The suspended soils from both sites exhibited differences in Verrucomicrobia, Proteobacteria, Firmicutes, Chloroflexi, and Actinobacteria.

Despite the unique environment of each suspended soil, formed by an individual plant or group of plants, the variation in microbial composition between the replicates was, but not very high at the phylum level. The strongest variations between the replicates were in the plantation terrestrial soil samples (Figure 3).

### 3.3. Significant Variation between Groups

In Table 2, we present the results of the pairwise comparisons of the samples. In comparing the two terrestrial soils, about 58% of the reads belonged to the OTUs, which abundance is variable between the samples. Comparison of the suspended soils from the forest and the ground forest soil revealed that sample FS2 was more similar to the ground soil, FG (22% of the reads from variable OTUs), while sample FS1 was less similar (59% from variable OTUs). The suspended soils from the plantation were similar to each other and the ground soil, PG (61–69% of the reads were from variable OTUs). For more detailed results, see Supplementary Figure S1.

### 3.4. Correlation Analysis

We performed a Mantel test to evaluate the correlations between the results of the Bray–Curtis dissimilarity beta metrics and our soil property measurements (partial for the normal mineral and suspended soils). Significant correlations were detected between beta diversity and basal respiration level (R = 0.42, *p*-value = 0.04). Possible reasons why we could not detect a more significant correlation include the high heterogeneity of soil microbiomes and the presence of microniches within the different microenvironments.

## 4. Discussion

The scale of epiphytic material accumulation in the studied habitats of Cat Tien [29] is comparable with published data [44,45]. The increase in epiphytic material mass has been significant over time. In the Canary Islands, it has increased from 0.5 kg/ha in an 8-year-old forest to 205 and 1253 kg/ha in a 25- and 60-year-old forest, respectively [44]. In a mountain rain forest in Monteverde, Costa Rica, the mass of epiphytic material in a 40-year-old secondary forest was 200 kg/ha, whilst in old-growth forest, it was two orders of magnitude greater, amounting to 33,100 kg/ha [45]. The largest mass of raw epiphytic material (44,000 kg/ha) was recorded in the Colombian mountain rainforests [15]. These data suggest that the epiphytic deposition of organic matter into tree crowns is a large-scale phenomenon, and similar to the accumulation of litter on the ground in temperate forests. Unlike temperate forests, tropical litter is quickly processed by the fauna, particularly termites, which absorb up to 50% of the litter [46,47]. This factor is likely key for the biodiversity in suspended soils, in which a huge bank of deposited organic matter is stored, which would rapidly disappear if it reached the ground under the trees.

The suspended soils associated with vascular epiphytes are a depository of unique microbiological biodiversity. This is consistent with earlier observations of the uniqueness of the biome communities associated with epiphytic consortia around tropical trees [48]. Suspended soils serve as a biodiversity depository, and function in a similar way to the upper horizons of more northern forest ground soils. This idea is confirmed by the fact that, on the whole, the biodiversity of suspended soils is greater than that of terrestrial soils in the tropics [17,18,19,20,49]. However, our studies have only partially confirmed this data. The alpha diversity metrics of the microorganisms reached a maximum in the native forests ground soils, being less in both the disturbed and suspended soils (Figure 1). The high diversity of native forest soil can be connected with the natural history of this location that has not suffered anthropological intrusion. This hypothesis is supported by the minimal variability in taxonomic composition of the replications from this site. The smaller amount of observed OTUs in the soils from the plantation sites can also be explained by the well-known fact that disturbed soils, as a rule, demonstrate smaller numbers of OTUs than undisturbed soils [50]. Moreover, the inter-repeat variation in the sample indices should be noted—this may be connected to the existence of tropical microniches with different nutritional regimes related to the environmental conditions.

Examination of the beta diversity reveals an even more complex structure (Figure 2). As with the alpha diversity, the forest ground soil samples (FG) formed a tight cluster, with one suspended soil sample (FS1) also forming a separate group. At the same time, the other suspended soil sample (FS2) spread by plot. According to the alpha diversity, the FS2 soil was the least diverse in the group of forest soils. Perhaps this over-variation can be explained by the existence of different taxonomic compositions between the replications. Soils from the plantation, both the ground soil (PG) and suspended soils (PS1 and PS2), clearly clustered together. This grouping can be reasonably explained by the variable and common components of the communities indicated by the pairwise analysis (Table 2). It is also interesting to note that two samples of suspended soils from different specimens of *D. quercifolia*, but from different habitats (FS1 and PS2), significantly differed in their microbiotas. This suggests that the specific composition of the microbiota is not the result of the plant formation but may be situational (associated with specific features of its distribution in a given place). The composition of prokaryotic phyla revealed in these soils, with the dominance of Acidobacteria, Proteobacteria, Verrucomicrobia and Actinobacteria in the upper horizons, has also been noted in other studies [22,23,24,25]. A high proportion of Acidobacteria is typically found in acidic soils [51,52]. Acidobacteriales (*Phylum Acidobacteria*) is capable of hydrolytic activity in relation to a wide range of biopolymers [53]. Solibacterales (*Phylum Acidobacteria*) possesses a large set of genes that are responsible for the mobilization of mineral phosphorus [54]. The ability to mobilize phosphorus may give these bacteria an advantage in red-yellow and alluvial soils, where mobile phosphorus is rare (Table 2). A high relative abundance of subgroup 1 Acidobacteria has been reported from Brazilian tropical forest [23] and savannah [55] soils. A high content of Acidobacteria is characteristic of many tropical soils [56]. In the terrestrial soils examined in this study, subgroup 2 dominated amongst the Acidobacteria in poor, non-volcanic soils (red-yellow and alluvial). It is likely that the Acidobacteria of subgroup 2 can be considered to be characteristic of the genesis of various acidic tropical soils that are poor in organic matter. It should be noted that, in general, our data on the terrestrial soils confirms and refines recently reported data on the microbiotas of terrestrial soils in the Cat Tien National Park [57].

A number of factors affect microbiome formation in suspended soils. It is beyond the scope of this work to determine the legacy of the suspended soils; however, according to our analysis, we can say that: (i) the taxonomic diversity in the suspended soils is less than that in the ground soils; and (ii) the taxonomic composition of the bacterial communities in the suspended soils of plants from the same species are not closer than the taxonomic compositions of the communities in the suspended soils of different plant species. It can be assumed that the species-specific effect of the epiphyte on the suspended soil has a much smaller effect on the microbiota than its location. However, to understand this accurately, a much larger sample is needed. In this sense, our data are consistent with a recent comparative study of the quantitative parameters of microbial diversity in the suspended soils of epiphytes from Borneo and Amazonia, where the authors noted significant differences between the microbial communities, suggesting that epiphytes created “convergent niches for microorganisms in tropical canopies” [58]. Moreover, we could not assess the heterogeneity of the microbiomes of the different soils in this study due to the fact that the Cat Tien Forest has been subjected to several different types of effects over the past century. However, it does indicate that the compositions of the microbiomes of terrestrial and suspended soils, along with the factor of microbial biodiversity formation, are complex and nonlinear.

## 5. Conclusions

Compared to the alpha biodiversity in epiphytic suspended soils, the highest bacterial diversity occurs in native forest ground soil. Plantation ground soil is less diverse, whilst the diversity of suspended soils is different, and less, than ground soil from same location. Interpretation of the beta diversity is very complicated. Most samples showed inter-replica variability. This may be connected to the high number of microniches in soils. The forest ground soil had minimal variation, perhaps due to the lack of disturbance and its historical legacy. The taxonomic composition at the phylum level was regular for soil bacterial communities, with some variations. In most of the pairwise comparisons to the OTUs, the abundances were significantly different, accounting for up to 60% of the reads. There were no significant differences in the comparison of the ground and suspended soils. We hypothesize that the taxonomic compositions of the bacterial communities in suspended soils of the same plants are not closer than the taxonomic compositions of the communities in the suspended soils of different plants.

## Figures and Tables

**Table 1:** Routine soil analysis results (average value ± standard deviation).

Sample Code	pH, H_2_O	BR, mg/g/h	Available Forms of Nutrients				N%	C%	C/N
			P_2_O_5_, mg/kg	K_2_O, mg/kg	N-NH_4_, mg/kg	N-NO_3_, mg/kg			
FG	4.99	0.11 ± 0.03	505 ± 15	1161 ± 45	115.6 ± 12	2.23 ± 0.07	2.21 ± 0.12	42.0 ± 0.87	19.0
FS1	5.77	0.09 ± 0.02	250 ± 12	2710 ± 56	66.3 ± 4	2.24 ± 0.12	1.36 ± 0.07	18.8 ± 0.12	13.8
FS2	5.53	0.06 ± 0.01	57 ± 3	668 ± 34	83.8 ± 7	7.87 ± 0.54	9.62 ± 0.05	41.4 ± 0.74	4.3
PG	5.99	0.03 ± 0.01	9 ± 2	70 ± 5	19.8 ± 2	<0.05	0.06 ± 0.02	0.21 ± 0.02	3.22
PS1	6.14	0.06 ± 0.01	117 ± 12	703 ± 46	62.5 ± 6	0.07 ± 0.02	0.14 ± 0.02	1.28 ± 0.07	9.04
PS2	5.68	0.10 ± 0.02	238 ± 10	2921 ± 76	157.5 ± 11	16.5 ± 1.20	1.68 ± 0.05	43.0 ± 0.81	25.5

**Table 2:** Results of the pairwise sample comparisons, including information about the abundance of taxa in the samples.

Sample Pair	Variable OTUs	Variable Reads	Common OTUs	Common Reads	% Variable OTUs	% Variable Reads
PS1–PS2	557	30,458	2525	18,486	18.1	62.2
PG–PS1	700	29,868	2443	19,076	22.3	61.0
PG–PS2	1002	34,063	2654	14,881	27.4	69.6
FS1–FS2	243	14,195	3054	28,631	7.4	33.1
FG–FS1	757	28,685	3116	20,259	19.5	58.6
FG–FS2	213	11,896	3300	30,930	6.1	27.8
FG–PG	857	28,359	3052	20,585	21.9	57.9

## Data Availability

DNA sequencing data associated with this work has been deposited at GenBank SUB7077111, BioProject ID: PRJNA609646, http://www.ncbi.nlm.nih.gov/bioproject/609646 (accessed on 11 May 2021).

## Supplementary Materials

The following are available online at https://www.mdpi.com/article/10.3390/microorganisms9051033/s1, Supplementary Table S1: The Spearman correlation coefficients for basal respiration and soil nutrient content, Supplementary Figure S1: Pairwise comparison of OTUs with significant changes in abundance (according DeSEQ2, p_adj < 0.05) between sample groups-order level (Log2FoldChange - estimated size effect for current OTUs, Order-taxonomic order for current OTUs).
