# Supplementary material for: Microbiomes in Suspended Soils of Vascular Epiphytes Differ from Terrestrial Soil Microbiomes and from Each Other
Source: Microorganisms. 2021 May 11;9(5):1033. doi: 10.3390/microorganisms9051033 (PMC8150705; doi:10.3390/microorganisms9051033)

Supplementary Table S1 *The Spearman correlation coefficients for basal respiration and soil nutrient content*

	**BR**	**P_2O_5**	**K_2O**	**NH_4**	**NO_3**	**Ntot**	**Ctot**
**BR**	1	0.91	0.86	0.78	0.03	0.31	0.23
**P_2O_5**	0.91	1	0.85	0.75	0.02	0.23	0.13
**K_2O**	0.86	0.85	1	0.81	0.23	0.33	0.37
**NH_4**	0.78	0.75	0.81	1	0.3	0.36	0.38
**NO_3**	0.03	0.02	0.23	0.30	1	0.64	0.67
**Ntot**	0.31	0.23	0.33	0.36	0.64	1	0.9
**Ctot**	0.23	0.13	0.37	0.38	0.37	0.9	1

Supplementary Fig. S1 *Pairwise comparison of OTUs with significant changes in abundance (according DeSEQ2, p_adj<0.05) between sample groups - order level (Log2FoldChange - estimated size effect for current OTUs, Order - taxonomic order for current OTUs).*

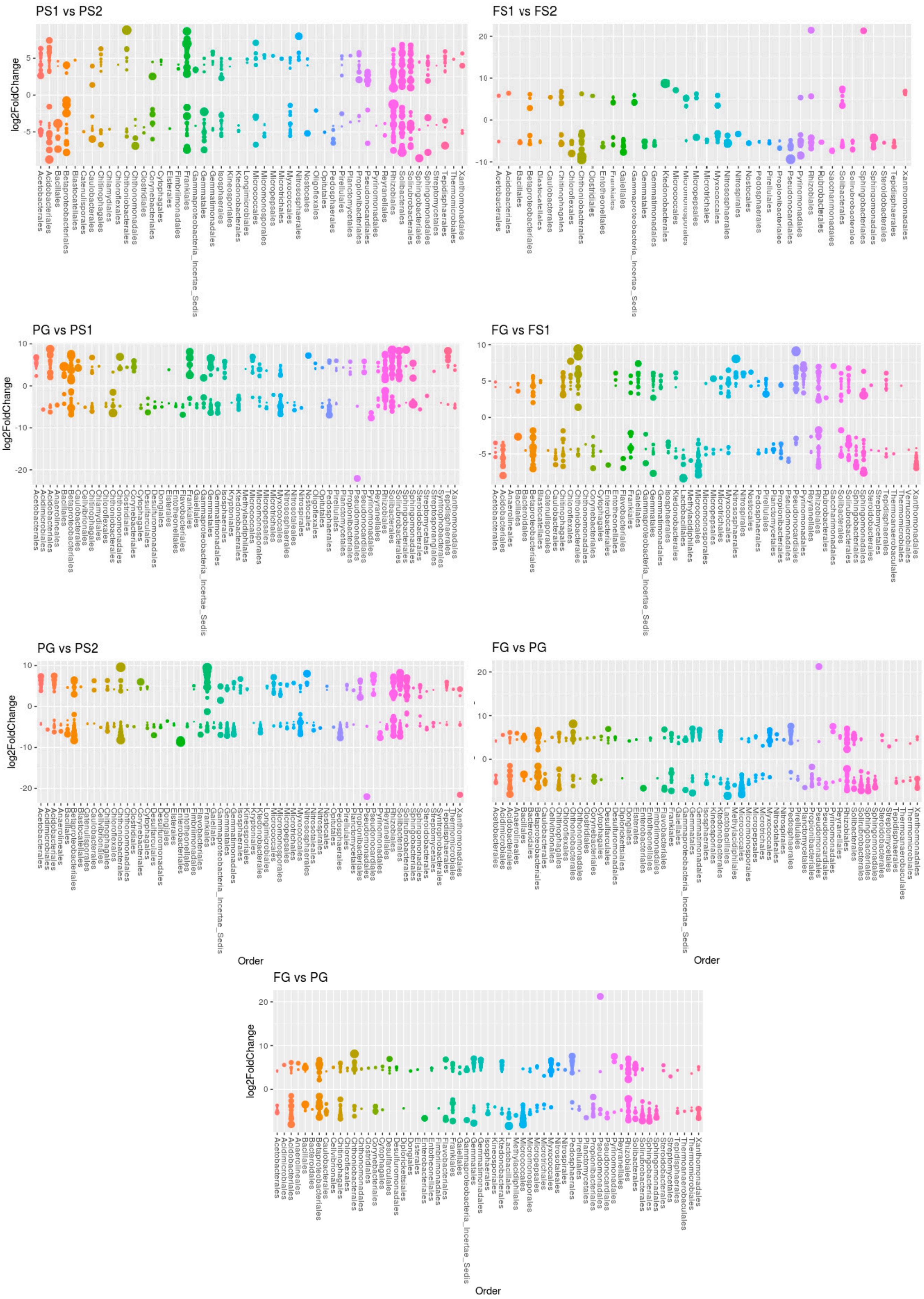

Supplement: Supplementary file 1 [file microorganisms-09-01033-s001.zip › microorganisms-1181359-supplementary.pdf]
